# Age-Adjusted D-Dimer in Ruling Out Acute Aortic Syndrome

**DOI:** 10.1155/2022/6864756

**Published:** 2022-02-05

**Authors:** Dayeon Lee, Yong Won Kim, Tae Youn Kim, Sanghun Lee, Han Ho Do, Jun Seok Seo, Jeong Hun Lee

**Affiliations:** Department of Emergency Medicine, Dongguk University Ilsan Hospital, Dongguk University College of Medicine, Goyang, Republic of Korea

## Abstract

**Background:**

Recently, D-dimer has been suggested as a biomarker to rule out acute aortic syndrome (AAS). Since it increases with age, this study was conducted to reveal whether an age-adjusted D-dimer can increase diagnostic accuracy in ruling out AAS.

**Method:**

A retrospective observational study design was used. Consecutive adult patients who visited an emergency room between January 2015 and September 2020 and who underwent a D-dimer test and computed tomography angiography for suspected AAS were enrolled. We calculated the diagnostic accuracy of both the conventional (0.5 *μ*g/ml) and age-adjusted (age × 0.01 in patients >50 years) D-dimer cut-offs.

**Result:**

D-dimer was higher in the AAS group (*n* = 82) than in the non-AAS group (*n* = 122) (10.85 (3.61–33.12) vs. 0.40 (0.23–1.07), OR: 1.139 (CI: 1.085 – 1.195), *p* < 0.001). The D-dimer plasma level had an area under the ROC curve of 0.915 (CI: 0.873–0.956) with AAS. At the age-adjusted cutoff point compared to a 0.5 *μ*g/ml cutoff, the sensitivity of 97.6% and the NLR of 0.04 did not change, but the specificity increased by 5.7% to 65.6%, the PPV increased by 3.6% to 65.6%, and the NPV slightly increased by 0.2% to 97.6%.

**Conclusion:**

Compared with a conventional method, the age-adjusted D-dimer cutoff may have higher specificity and PPV while maintaining high sensitivity for ruling out AAS.

## 1. Introduction

Acute aortic syndrome (AAS) is a life-threatening cardiovascular emergency requiring early diagnosis and includes acute aortic dissection (AD), intramural hematoma (IMH), penetrating aortic ulcer (PAU), and aneurysmal rupture [1, 2]. However, since AAS has common and unspecific symptoms, it is not easy to differentiate it from other diseases by simple primary evaluation without advanced imaging tests such as contrast-enhanced tomography angiography (CTA), which is the most frequently used [3]. However, CTA needs to be performed with proper selection because it has additional costs, carries risks (such as radiation exposure and contrast-induced nephropathy), and has a low positivity rate for suspected AAS [4]. Therefore, a simple and quick laboratory test to rule out AAS would be of great value. D-dimer is a degradation product of crosslinked fibrin and has been widely used as a screening biomarker for acute pulmonary embolism, and its clinical value for ruling out AAS has recently been established [5]. Since the plasma concentration of D-dimer can increase with age, it is known that the use of an age-adjusted D-dimer is more specific than the standard threshold (0.5 *μ*g/mL) in ruling out acute pulmonary embolism [6, 7]. However, there are still few studies on the clinical efficacy of age-adjusted D-dimer in AAS [8]. Therefore, this study was conducted to evaluate the diagnostic accuracy of age-adjusted D-dimer compared with the current standard threshold in AAS.

## 2. Subjects and Methods

### 2.1. Study Design and Hospital Setting

This retrospective observational study was conducted with consecutive patients older than 18 years with suspected AAS who underwent aorta CTA in the emergency department of an academic tertiary care center from January 2015 to September 2020. Patients were excluded if they were in a trauma-related condition, had prior AAS, or did not obtain a D-dimer plasma level from initial laboratory blood test.

During the study period, aortic surgery was available for 24 hours at our institution by thoracic surgeons. If the aortic dissection detection risk score (AAD-RS) was ≥1 or the clinician suspected an aortic emergency (acute aortic syndrome or traumatic aortic injury) among patients who visited the emergency department, D-dimer plasma levels were routinely measured at the time of initial blood sampling, and an aorta CTA scan was performed under the direction of the attending emergency physicians for differential diagnosis. D-dimer levels were measured by Sysmex CS-5100 (Sysmex, Kobe, Japan) with the quantitative automated immunoturbidimetric assay. The aorta CTA scan protocolized with a range of 1 cm superior to lung apices through aortic bifurcation (level of S1), was performed with a SOMATOM Definition 64-slice CT Scanner (Siemens, Erlangen, Germany), and the aorta CTA was read by a radiologist.

### 2.2. Data Collection and Study Definition

Clinical data obtained from electronic medical records, included age, gender, body mass index (BMI), social and past medical history (hypertension, diabetes mellitus, and smoking), initial clinical presentation (chief complaint, symptom onset time, initial vital signs, and aortic dissection detection risk score (AAD-RS)), initial (sampled at least within 1 hour after emergency department visit) plasma D-dimer level (*μ*g/mL), final diagnosis, and mortality. If the D-dimer value was reported as <0.23 or >33.60 *μ*g/mL by the setting of laboratory equipment, it was entered as 0.23 or 33.60 *μ*g/mL at the time of data collection. Two emergency medicine physicians independently reviewed the data.

BMI was calculated as weight (kilograms) divided by the square of height (meters). Both current smokers and exsmokers were defined as smokers. An initial systolic BP (SBP) less than 90 mmHg or diastolic BP (DBP) less than 60 mmHg after an ER visit was defined as low BP. Mortality was defined as death during hospital admission or moribund discharge.

The ADD-RS was retrospectively calculated according to the presence or absence of risk markers classified from three aortic dissection detection (ADD) risk categories (predisposing conditions, pain features, and physical findings) in the medical records, as suggested by the 2010 AHA guidelines [1]. The predisposing conditions were as follows: history of Marfan syndrome or of other connective tissue disease, family history of aortic disease, history of known aortic valve disease, history of recent aortic manipulation, and history of known thoracic aortic aneurysm. The pain features were as follows: abrupt onset of pain, severe pain intensity, and ripping or tearing quality of pain. For charts reporting a pain scale, the severity of pain was defined as a numeric rating scale of 7–10. The physical findings were as follows: pulse asymmetry or systolic blood pressure differential (>20 mm Hg) between extremities, focal neurological deficit (altered mentality, dysarthria, side weakness, acute paraplegia, disequilibrium, Horner syndrome, and hoarseness), new murmur of aortic insufficiency, and shock state or hypotension (systolic blood pressure ≤90 mm Hg). The ADD-RS was calculated based on the number of categories where at least one risk marker was present.

The cut-off value of D-dimer to rule out AAS was set to 0.5 *μ*g/ml or age-adjusted D-dimer (0.5 *μ*g/ml in patients under 50 years and age × 0.01 in patients 50 years or older) was carried out previously for pulmonary embolism [7]. Any of the following diagnoses by aorta CTA were considered as AAS: AD, IMH, PAU, and rupture of aortic aneurysm. The patients were divided accordingly into AAS or non-AAS groups.

### 2.3. Statistical Analysis

We compared the study variables of the AAS and non-AAS groups. Continuous variables are reported as median values (interquartile range, IQR) and were compared by the Mann–Whitney test. Nominal data were calculated as percentages based on the frequency of occurrence and compared using the chi-squared or Fisher's exact test, as appropriate. Multivariate logistic regression analysis was used to associate the single variables with AAS. The resulting odds ratios (ORs) are presented with 95% confidence intervals (95% CIs). To assess the diagnostic performance of D-dimer measurements, the area under the receiver operating characteristic (ROC) curve was calculated for AAS with the control group. The sensitivity, specificity, predictive values, and likelihood ratios of D-dimer at the cut-off level were evaluated. A two-sided *p*value of less than 0.05 was considered statistically significant. Analyses were performed using the IBM Statistical Package for the Social Sciences (SPSS) software version 24.0 (SPSS, Inc., Chicago, IL, USA).

## 3. Results

During the study period, 301 adult patients with suspected aortic emergencies were admitted to the emergency department and underwent aorta CTA. Among them, 33 cases were excluded because of trauma-related conditions, 43 were excluded because of prior AAS, and 21 were excluded because D-dimer plasma levels were not measured. Finally, 204 patients were enrolled for the analysis. These were divided into two groups: 122 (59.8%) in the non-AAS and 82 (40.2%) in the AAS groups, respectively.

**Figure 1:**
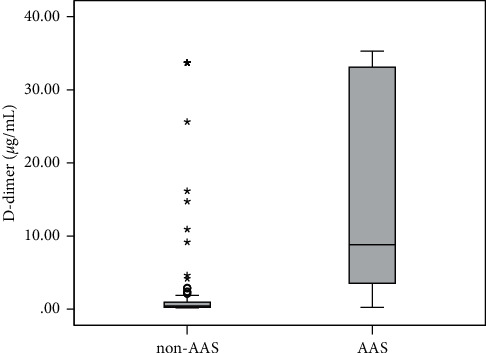
Box plot for D-dimer plasma levels between the ASS and non-ASS groups (AAS, acute aortic syndrome).

The patient characteristics, including the clinical factors associated with each group, are shown in Table 1. The AAS group had a higher median age (70 (59–82) vs 63 (55–77), *p*=0.017), more past history of hypertension (64.6% vs. 46.7%, *p*=0.015), lower SBP (137 (111–163) vs. 149 (131–170) mmHg, *p*=0.001), lower DBP (74 (61–87) vs. 85 (73–99) mmHg, *p*=0.001), more low BP at initial vital sign (26.8% vs. 9.0%, *p*=0.001), higher ADD-RS (2 (1–2) vs. 1 (1–1), *p* < 0.001), and higher discharge mortality (23.2% vs. 4.9% *p* < 0.001) than those in the non-ASS group (Table 1). The D-dimer plasma levels were higher in the AAS group than non-AAS group (10.85 (3.61–33.12) vs. 0.40 (0.23–1.07) *μ*g/mL, *p* < 0.001) (Figure 1). There was no significant difference in other clinical variables (gender, body mass index, diabetes, smoker, smoking duration, chief complaint, initial heart rate, and initial body temperature) between the two groups (Table 1).

Multivariate logistic regression revealed that factors associated with acute aortic syndrome were male gender (OR: 2.946 (95% CI: 1.275–6.806), *p*=0.011), past history of hypertension (OR: 3.032 (95% CI: 1.313–7.000), *p*=0.009), ADD-RS (OR: 2.852 (95% CI: 1.486–5.472), *p*=0.002), and D-dimer concentration (*µ*g/mL) (OR: 1.139 (95% CI: 1.085 = 1.195), *p* < 0.001) (Table 2).

**Figure 2:**
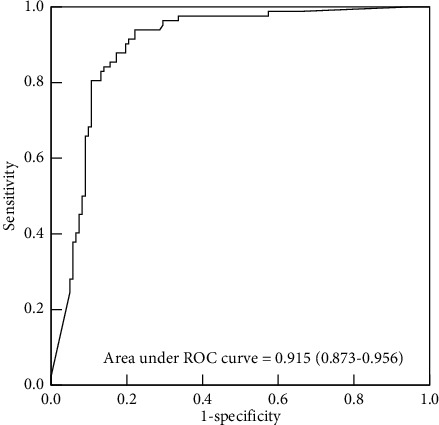
Receiver operating characteristic curve of D-dimer for the detection of acute aortic syndrome.

The D-dimer plasma level had an area under the ROC curve of 0.915 (CI: 0.873–0.956), with a higher D-dimer level indicating AAS (Figure 2). At the cut-off value of 0.5 *µ*g/mL for ruling out ASS, sensitivity of 97.6% and specificity of 59.8%, positive predictive value (PPV) of 62.0%, negative predictive value (NPV) of 97.3%, positive likelihood ratio (PLR) of 2.4, and negative likelihood ratio (NLR) of 0.04 were presented. At the age-adjusted cutoff point from the original cutoff of 0.5, the sensitivity and NLR did not change, but the specificity increased by 5.7% to 65.6%, the PPV increased by 3.6% to 65.6%, the PLR increased by 0.4 to 2.83, and the NPV slightly increased by 0.2% to 97.6% (Table 3).

There were two patients who presented false-negative results in both D-dimer approaches (cut-off of 0.5 and age-adjusted) for the ruling out of AAS; one patient with IMH and one patient with PAU.  Table 4 summarizes the two patients with false-negative acute aortic syndrome.

The D-dimer levels for each of the detailed diagnoses identified between the AAS group and the non-AAS group are shown in Table 5. In the AAS group, the order in which the median values of D-dimers were higher was as follows: aortic aneurysm rupture (16.78 (6.7–33.7)), AD (13.62 (3.61–33.70)), IMH (5.83 (3.30–14.4)), and PAU (1.97 (1.10–17.84)). In the non-AAS group, the order in which median D-dimers were higher, was sepsis (3.69 (1.57–15.45)), malignancy (1.25 (0.72–1.74)), and visceral vascular thrombus or focal dissection (0.81 (0.23–2.60)), and the median value of D-dimer did not exceed 0.5 *μ*g/mL in other diseases (Table 5).

## 4. Discussion

We confirmed that D-dimer is useful as an initial laboratory marker to rule out AAS, as others have found [5, 9]. The D-dimers are typical degradation products of cross-linked fibrin found in the circulation, and elevated D-dimer levels can generally be seen as secondary fibrinolysis and intravascular activation of the coagulation system under various conditions, including AAS [10]. In previous studies, the diagnostic accuracy for AAS at the cut-off value of 0.5 *μ*g/mL was reported to have a sensitivity of 95% (95% CI: 90–98%) and a specificity of 60% (95% CI: 49–71%), and this result was similar to our data (sensitivity of 98% and specificity of 60%) [5].

In addition, we found that specificity (from 60 to 66%) and PPV (from 62 to 66%) can be increased while maintaining high sensitivity (98%) when using the age-adjusted D-dimer approach, compared to using the standard fixed cut-off (0.5 *µ*g/mL) to rule out AAS. Because D-dimer increases with age, the standard approach may have limited clinical usefulness in elderly patients [6, 10]. Therefore, in order to offset this and increase the diagnostic value of the biomarker, age-adjusted for D-dimer has been recently recommended in the ruling out of pulmonary embolism or deep vein thrombosis [7, 11]. In regards to AAS, however, there are few studies on the usefulness of age-adjusted D-dimer [8, 12, 13]. Kotani et al. reported that using age-adjusted D-dimer for ruling out AAS increased specificity (from 44% to 58%) without change in sensitivity (97%), in a retrospective observational study of adult patients with chest pain [8]. Similarly, Morello et al. reported that using age-adjusted D-dimer for ruling out AAS increased specificity (from 64% to 71%) and slightly decreased sensitivity (from 97% to 95%) in a multicenter prospective observational study of adult patients who needed to rule out AAS [12, 13]. In their studies, the tendency of specificity and sensitivity parameters according to each approach were similar to our findings, but the difference was that the specificity change was in a different range. This may be because of the study population which differed compared to our study population in which CTA was performed for evaluation of suspected AAS.

Other factors related to AAS that were revealed in our results included being male, a history of hypertension, and ADD-RS. Male gender and hypertension are well-known risk factors for AAS [14]. However, it would be difficult to simply use these factors to rule out AAS as it would result in too many false negatives. As well, it is acceptable that the risk of AAS increases as ADD-RS increases. However, it remains questionable to rule out AAS by ADD-RS alone because up to 4.3% or 36.5% of AD show low (0) or even intermediate risk (1) scores [15]. Therefore, some studies have suggested the efficacy of combining ADD-RS with D-dimer (ADD-RS ≤1 plus D-dimer <0.5 *μ*g/mL) in ruling out ASS. Sensitivities of 94–99% and specificities of 57–63% with this approach were reported [13, 16]. In addition, in a meta-analysis study that included 4 studies on the diagnostic rule out of suspective AAS through ADD-RS plus D-dimer, age-adjusted D-dimer with ADD-RS≤1 has a 5∼10% increase in specificity and a 1∼2% decrease in sensitivity compared to the standard cut-off with ADD-RS ≤1 [17]. During the study period, confirmative aorta CTA images were mostly performed in ADD-RS ≥ 1 in our hospital setting, with only a few exceptions for 0 scores (*n* = 7), and these were according to the subjective judgment of the clinician. Therefore, we did not perform a subgroup analysis of the diagnostic value of D-dimer according to ADD-RS. Further research will be needed to reveal the diagnostic accuracy of the combination of age-adjusted D-dimer and ADD-RS to rule out AAS.

### 4.1. Limitations

This study has some limitations. First, as a retrospective design rather than a confirmative study, there may be missing data in the analysis, such as past medical history or physical findings, because data collection was not carried out according to a prior protocol. Second, as a single-center study, regional patient characteristics and the medical environment may affect the diagnostic rate and prevalence. Third, there may be selection bias in this study. This is because aortic CTA may not have been performed according to clinician decision in some patients with low AAS risk, and some patients may have refused to proceed with aortic CTA. Finally, there might be ASS cases that are not included in the study because they were diagnosed by other confirmative images including transesophageal echocardiography (TEE), magnetic resonance angiography (MRA), interventional angiography, chest CT, and abdominal CT. However, TEE, MRA, or interventional angiography were not routinely performed as a screening tool for diagnosing AAS at our institution during the study period. And if AAS is diagnosed incidentally on chest CT or abdominal CT and is included in the study, it is difficult to establish a control group.

## 5. Conclusions

Using an age-adjusted D-dimer for ruling out AAS is as safe as using a standard D-dimer cut-off in emergency department patients with suspected AAS. Using an age-adjusted D-dimer may rule out more AAS diagnoses by increasing diagnostic accuracy (especially in specificity and PPV) than using the fixed D-dimer cutoff of 0.5 *μ*g/mL. The use of D-dimer may be limited in some subtypes of AAS such as intramural hematomas and penetrating aortic ulcers. Additional prospective trials are needed to confirm the results of this study and to determine whether an age-adjusted cutoff can improve cost-effectiveness or quality of care.

## Figures and Tables

**Table 1:** Comparison of general characteristics between the AAS and non-AAS groups.

	Total (*n* = 204)	Non-AAS (*n* = 122)	AAS (*n* = 82)	*p*value
Age (yrs)	67 (56–80)^*∗*^	63 (55–77)^*∗*^	70 (59–82)^*∗*^	0.017
Male gender, no. (%)	123 (60.3)	67 (54.9)	56 (68.3)	0.060
Body mass index	24.5 (22.2–26.8)^*∗*^	24.6 (22.1–27.2)^*∗*^	24.4 (22.4–26.6)^*∗*^	0.871
Medical history				
Diabetes, no. (%)	57 (27.6)	36 (29.5)	21 (25.6)	0.634
Hypertension, no. (%)	110 (53.9)	57 (46.7)	53 (64.6)	0.015
Smoker, no. (%)	77 (37.7)	41 (33.6)	36 (43.9)	0.144
Pack∙years among smokers	30 (20–40)^*∗*^	30 (20–40)^*∗*^	29 (18–40)^*∗*^	0.868
Clinical presentation				
Chief complaint				
Chest pain, no. (%)	113 (55.4)	74 (60.7)	39 (47.6)	0.367
Back pain, no. (%)	24 (11.8)	15 (12.3)	9 (11.0)	
Abdominal pain, no. (%)	22 (10.8)	10 (8.2)	12 (14.6)	
Radiating pain to extremity, no. (%)	3 (1.5)	1 (0.8)	2 (2.4)	
Neurologic deficit, no. (%)	15 (7.4)	7 (5.7)	8 (9.8)	
Syncope or presyncope, no. (%)	7 (3.4)	3 (2.5)	4 (4.9)	
Other symptoms	20 (9.8)	12 (9.8)	8 (9.8)	
Onset to visit interval (hrs)	4.0 (1.5–46.3)^*∗*^	5.7 (1.4–54.0)^*∗*^	3.5 (1.8–7.0)^*∗*^	0.050
Initial vital sign				
SBP (mmHg)	144 (121–168)^*∗*^	149 (131–170)^*∗*^	137 (111–163)^*∗*^	0.001
DBP (mmHg)	81 (65–97)^*∗*^	85 (73–99)^*∗*^	74 (61–87)^*∗*^	0.001
SBP <90 mmHg or DBP <60 mmHg, no. (%)	33 (16.2)	11 (9.0)	22 (26.8)	0.001
HR (rate/min)	80 (68–89)^*∗*^	80 (72–92)^*∗*^	80 (67–88)^*∗*^	0.824
BT (°C)	36.5 (36.1–36.7)^*∗*^	36.5 (36.3–36.7)^*∗*^	36.5 (36.2–36.7)^*∗*^	0.132
ADD-RS	1 (1–2)^*∗*^	1 (1–1)^*∗*^	2 (1–2)^*∗*^	<0.001
D-dimer (*μ*g/mL)	1.60 (0.34–10.85)^*∗*^	0.40 (0.23–1.07)^*∗*^	10.85 (3.61–33.12)^*∗*^	<0.001
Mortality	25 (12.3)	6 (4.9)	19 (23.2)	<0.001

^*∗*^Median (interquartile range); AAS, acute aortic syndrome; SBP, systolic blood pressure; DBP, diastolic blood pressure; HR, heart rate; BT, body temperature; ADD-RS, aortic dissection detection risk score.

**Table 2:** Multivariate analysis of clinical factors associated with acute aortic syndrome.

Clinical factors	Odds ratio	95% CI	*p*value
Age (yrs)	1.005	0.977–1.033	0.739
Male gender	2.946	1.275–6.806	0.011
Past history of hypertension	3.032	1.313–7.000	0.009
Symptom onset to visit interval (hr)	1.000	0.999–1.001	0.626
Low BP at ER visit	0.976	0.335–2.840	0.964
ADD-RS	2.852	1.486–5.472	0.002
D-dimer (*μ*g/mL)	1.139	1.085–1.195	<0.001

CI, confidence interval; BP, blood pressure; ER, emergency room; ADD-RS, aortic dissection detection risk score.

**Table 3:** Comparison of diagnostic accuracy between D-dimer cutoff of 0.5 *μ*g/mL and age-adjusted D-dimer for ruling out acute aortic syndrome.

Diagnostic variables	D-dimer of 0.5 *μ*g/mL	Age-adjusted D-dimer
Sensitivity (%)	97.56 (91.47–99.70)^*∗*^	97.56 (91.47–99.70)^*∗*^
Specificity (%)	59.84 (50.58–68.61)^*∗*^	65.57 (56.43–73.94)^*∗*^
Positive predictive value (%)	62.02 (56.73–67.03)^*∗*^	65.57 (59.80–70.92)^*∗*^
Positive likelihood ratio	2.43 (1.95–3.02)^*∗*^	2.83 (2.21–3.63)^*∗*^
Negative predictive value (%)	97.33 (90.21–99.31)^*∗*^	97.56 (91.00–99.37)^*∗*^
Negative likelihood ratio	0.04 (0.01–0.16)^*∗*^	0.04 (0.01–0.15)^*∗*^

^*∗*^95% confidence interval.

**Table 4:** Clinical presentation of two cases of false negative results obtained with the D-dimer approach.

Case	Clinical description	Symptom onset	ADD risk factors	ADD-RS	Chest X-ray finding	D-dimer	Type of AAS
1	56/M, anterior and posterior chest pain, history of hypertension, diabetes mellitus, smoking, high blood pressure (SBP 171/ DBP113) at ER visit	0.5 hr ago	Sudden, severe pain	1	Normal mediastinum	0.34 *μ*g/mL	IMH, descending thoracic aorta
2	61/M, anterior chest pain, history of diabetes mellitus	1 hr ago	Sudden, severe, ripping pain	1	Normal mediastinum	0.23 *μ*g/mL	PAU, descending thoracic aorta

ER, emergency room; ADD, aortic dissection detection; ADD-RS, aortic dissection detection risk score; ASS, acute aortic syndrome; IMH, intramural aortic hematoma; PAU, penetrating aortic ulcer.

**Table 5:** D-dimer (*μ*g/mL) levels in diagnosis categories.

Diagnosis	No. (%)	D-dimer^*∗*^
AAS	82	
Aortic dissection	46 (56.1)	13.62 (3.61–33.70)^*∗*^
IMH	19 (23.2)	5.83 (3.30–14.4)^*∗*^
PAU	3 (3.7)	1.97 (1.10–17.84)^*∗*^
Rupture of aortic aneurysm	14 (17.1)	16.78 (6.7–33.7)^*∗*^
Non-AAS	122	
Acute myocardial infarction	22 (18.0)	0.41 (0.23–1.18)^*∗*^
Angina or other coronary disease	19 (15.6)	0.26 (0.23–0.37)^*∗*^
Other heart disease	8 (6.6)	0.50(0.31–0.75)^*∗*^
Other visceral vascular thrombus or focal dissection	12 (9.8)	0.81(0.23–2.60)^*∗*^
Sepsis, infectious disease	8 (6.6)	3.69 (1.57–15.45)^*∗*^
Gastrointestinal disease	12 (9.8)	0.33 (0.23–0.74)^*∗*^
Malignant disease	7 (5.7)	1.25 (0.72–1.74)^*∗*^
Musculoskeletal disorder	12 (9.8)	0.29 (0.23–0.66)^*∗*^
Neuropsychiatric condition	2 (1.6)	0.23
Pneumothorax	1 (0.8)	0.23
Ureter stone	1 (0.8)	0.23
Uncertain cause	18 (14.8)	0.42 (0.23–0.52)^*∗*^

^*∗*^Median (interquartile range); ASS, acute aortic syndrome; IMH, intramural aortic hematoma; PAU, penetrating aortic ulcer.

## Data Availability

The data used to support the findings of this study are available from the corresponding author upon request.
